# Sexuality and Sexual and Reproductive Health Depiction in Social Media: Content Analysis of Kinyarwanda YouTube Channels

**DOI:** 10.2196/46488

**Published:** 2023-09-27

**Authors:** Thierry Claudien Uhawenimana, Marie Grace Sandra Musabwasoni, Richard Nsengiyumva, Donatilla Mukamana

**Affiliations:** 1 Department of Midwifery School of Nursing and Midwifery, College of Medicine and Health Sciences University of Rwanda Kigali Rwanda; 2 Department of Mental Health School of Nursing and Midwifery, College of Medicine and Health Sciences University of Rwanda Kigali Rwanda

**Keywords:** sexuality, sexual and reproductive health, Kinyarwanda YouTube channels, content analysis, social media, media platform, COVID-19

## Abstract

**Background:**

Social media platforms such as YouTube can be used to educate people of reproductive age about healthy and nonrisky sexual and reproductive health (SRH) practices and behaviors. However, there is a paucity of evidence to ascertain the authenticity of sexuality and SRH content on Kinyarwanda YouTube, making it difficult to determine the extent to which these topics are covered, the characteristics of available videos, and the themes covered by these videos.

**Objective:**

The aims of this study were (1) to determine the extent to which YouTube channels in Kinyarwanda-language videos address sexuality and SRH issues, identify the general characteristics of the available videos (type of video, when published, intention for the audience, and content focus), and the aspects of sexuality and SRH covered; and (2) to identify the themes covered by retrieved Kinyarwanda videos, and the extent to which the channels have been used to communicate issues of sexuality and SRH during the COVID-19 pandemic.

**Methods:**

Using a content analysis approach, we searched Kinyarwanda YouTube channels to analyze videos about sexuality and SRH. The adopted framework for data collection from social media platforms builds on three key steps: (1) development, (2) application, and (3) assessment of search filters. To be included, an audio and/or visual video had to be in Kinyarwanda and the video had to be directed to the general public. Descriptive statistics (frequency and percentages) were computed to characterize the basic characteristics of retrieved channels, portrayal of the videos, and presentation of sexuality and SRH themes that emerged from retrieved videos. Further analysis involved cross-tabulations to explore associations between the focus of the channel and the date when the channel was opened and the focus of the channel and who was involved in the video.

**Results:**

The YouTube search retrieved 21,506 videos that tackled sexuality and SRH topics. During the COVID-19 pandemic, there was a 4-fold increase (from 7.2% to 30.6%) in channels that solely focused on sexually explicit content. The majority of the 1369 retrieved channels (n=1150, 84.0%) tackled the topic of sexuality, with sexually explicit content predominantly found in the majority of these videos (n=1082, 79%), and only 16% (n=287) of the videos covered SRH topics.

**Conclusions:**

This is the first study to analyze the use of YouTube in communicating about sexuality and SRH in the Kinyarwanda language. This study relied on videos that appeared online. Further research should gather information about who accesses the videos, and how channel owners and individuals involved in the videos perceive the impact of their videos on the Rwandan community’s sexuality and SRH.

## Introduction

The World Health Organization emphasizes that people should have access to comprehensive, good-quality information about sex and sexuality in terms of assuring universal health coverage [1]. In the context of this study, sexuality refers to an individual’s capacity to experience and express their sexual feelings such as love, lust, friendship, attraction, and dating [2]. Sexuality also refers to the way people express their sexual feelings in relation to their gender identity, sexual orientation, eroticism, pleasure, intimacy, and reproduction in thoughts, fantasies, desires, beliefs, attitudes, values, behaviors, practices, roles, and relationships [2,3]. Sexual and reproductive health (SRH) refers to safe sex practices, including, but not limited to, prevention of sexually transmitted infections, condom use, pregnancy, childbirth, family planning, fertility, adolescent SRH, abortion, and maternal health.

To optimize people’s knowledge of sexuality and SRH matters, among available communication approaches, social media platforms can be used as a means to reach people of reproductive age and educate them about healthy and nonrisky sexuality and SRH practices and behaviors [4,5]. When used effectively, social media can illuminate young people’s sexuality and SRH concerns such as condom use, birth control, and sexually transmitted infections and safe sex practices [6,7]. However, social media can also lead to harmful impacts on people’s SRH. Research suggests that some adolescents and young adults may be at a high risk of sexual behaviors such as sexualized text communication (sexting), exposure to pornography, online dating, or other risk-taking behaviors associated with social media use [8,9]. It is also suggested that victims of cyber bullying and those who frequently misuse social media for SRH matters may be at increased risk of sexually transmitted infections and unplanned pregnancy [8].

There are several social media platforms available, including those used for chatting and those used to stream videos. Among social streaming video media platforms, YouTube is the most popular platform with over 1 billion users globally [10,11]. Unless the videos uploaded contain racial and other information deemed to be sensitive to potential viewers, within the normal circumstances, YouTube does not question the credibility of video uploads [11,12]. Thus, inappropriate health information lacking evidence can often be posted by some users on their channels [7,13]. One of the downsides of YouTube is that some of the uploaded content may not have been checked for authenticity; therefore, registered users can post any content they choose for the viewers to access freely [11].

In the context of sexuality and SRH information, there is promising evidence that social media can serve as an effective sexual health promotion intervention to change young people’s sexual behaviors and reduce the number of positive chlamydia and gonorrhea cases [7]. However, a review of the evidence suggested that more than 80% of young people in Sub-Saharan African countries may be victims of sexual, gender-based, and other forms of violence perpetrated on some social media platforms [14]. Moreover, a study commissioned by Women Deliver International revealed that adolescent girls and young women in Rwanda and Malawi are turning to digital platforms as a one-stop shop, where they look for information about their bodies, health, and relationships [15]. Various digital platforms are used to look up sexuality and SRH information, with Google and YouTube being the most preferred online platforms in India and Rwanda [15]. In Malawi, adolescents searched for information on contraception, sexual health, and abortion, whereas in Rwanda, they primarily searched for information on love, relationships, and puberty [15]. This multinational study found that adolescent girls and young women sought sensitive SRH information through internet-enabled phones because this modality can afford privacy and/or anonymity [15]. However, this study left gaps in evidence regarding the content of sexuality and SRH information found on the digital social media platforms, the owners of such platforms, and messages conveyed in the content uploaded on these media. Hence, the information from this study is not sufficient to obtain the real picture of social media use in communicating about sexuality and SRH in these settings.

With the current promotion of technology use and the smartphone penetration on the Rwandan market, there has been a surge in the number of people using social media. As of January 2021, there were 4.12 million internet users, among whom 850,000 were social media users [16]. This increase has been instigated by the Rwandan government’s policies and initiatives aiming at encouraging the use of new media technologies in various sectors to speed up the delivery of services [16]. With this rapid increase of internet use, there is a likelihood that social media platforms may facilitate users to provide misleading and sensitive information about sexuality and SRH. However, since there is a paucity of studies that focused on the application of social media platforms to communicate sexuality and SRH issues, we cannot ascertain the authenticity of sexuality and SRH on social media platforms, especially YouTube, to understand the extent to which sexuality and SRH are covered, characteristics of available videos, and themes covered by those videos. Moreover, no study has analyzed the content in Kinyarwanda-language (the native language of the Rwandan people) YouTube channels to examine the specific sexuality and SRH topics that are uploaded onto this platform.

Therefore, the aims of this study were to determine the extent to which YouTube channels in the Kinyarwanda language cover videos about sexuality and SRH issues, identify the general characteristics of the available videos (type of video, when published, intention for the audience, and content focus), and the aspects of sexuality and SRH covered by the retrieved videos. Furthermore, we sought to identify the themes covered by the retrieved Kinyarwanda videos, and the trend with which the YouTube video channels have been used to communicate about sexuality and SRH during the COVID-19 pandemic.

## Methods

### Design

This study analyzed the content of Kinyarwanda-language videos about sexuality and SRH posted on YouTube. No year limit was set. We searched YouTube from July 11, 2021, to January 25, 2022. The study adapted the framework proposed by Kim et al [17], which outlines three steps to follow in collecting data from social media platforms: (1) development of search filters, (2) application of search filters, and (3) assessment of the search filters [17]. First, search terms are identified and then filters are set to build a search string that will enable the retrieval of relevant social media posts. Second, the search terms developed are used to search the social media platform of interest. Third, an assessment of the search strategy is performed to determine its relevance and robustness. For the purpose of this study, this framework [17] served as a guide for the data collection process, mainly in developing and applying search filters.

### Search Approach

Videos were included if they focused on sexuality issues such as sexual pleasure and performance, sexual education, and sexual dysfunction. Videos were also included if they tackled SRH issues, including, but not limited to, fertility, sexually transmitted diseases, adolescent health, pregnancy, childbirth, family planning, and abortion. The following Kinyarwanda key terms were used first in combination: *imibonano mpuzabitsina* (sexual intercourse), *gusama* (getting pregnant), *ubuzima bw’imyororokere* (reproductive health), *kuboneza urubyaro* (family planning), *gukuramo inda* (abortion), *igituba* (vagina), *imboro* (penis), *igitsina* (sex), *kurongora* (making love), *indwara zandurira mu mibonano mpuzabitsina* (sexually transmitted diseases/infections).

These keywords were first searched using Boolean logic “AND” and “OR” connectors. Each phrase and/or word, including their formal and informal equivalents where available in Kinyarwanda language, were then individually applied to search YouTube. A manual search of some of the obtained channel names was applied to track other potential channels publishing the same content in the Kinyarwanda language. The search for channels was performed three times a week to identify any new channel that had uploaded content related to the subject under study. To access all videos about sexuality and SRH in the Kinyarwanda language, including those that might have flagged content, we logged in to the specific accounts. During the first five searches, all outcomes were screened to identify videos relevant to the study. In subsequent searches, the upload date filter was applied to screen only videos added to the channels within 1 month, 1 week, and the last day, depending on the previous time the search was carried out. Once a channel had some videos about sexuality and SRH, they were coded and added to the list of considered videos.

### Eligibility Criteria

We included all types of videos, either visual or audio, regardless of their length. To be included, an audio and/or visual video covering sexuality and SRH topics had to be in Kinyarwanda and the video had to be directed to the general public by the uploader’s default setting. A video was excluded if it had no audio talk component and if it was not in the Kinyarwanda language. Videos that did not focus on sexuality and SRH messages were excluded from the analysis.

### Data Collection and Coding Scheme

Given that this study dealt with sensitive content, the researchers set up YouTube accounts to enable using YouTube privacy features when watching the retrieved videos. Data collection entailed two steps: (1) collection of the background information about the video in relation to the topic and (2) evaluation of retrieved videos to generate the content focus of the videos in relation to sexuality and SRH matters.

A data set was developed in SPSS software, which is commonly used to manage data, perform basic and advanced statistical analyses, and for data documentation in social and health sciences research [18]. The SPSS data set was used to record information about the retrieved channels and videos. We collected the YouTube channels’ basic information, including the video title, video length, opening date, posting timeline, number of views, number of subscribers, sex (male/female) of the narrator of the content about sexuality and SRH and their behavior while narrating, how channels protect viewers against any sensitive videos and information covered in the videos, and the type of language (colloquial, formal, or professional) used in the videos. Additional information included the proprietor of the channel and whether the information uploaded on the channel was delivered by an expert or a trained professional in the field of sexuality and SRH. Additional analysis involved examining the opening time and posting date to determine if the opening time matched the video posting trends.

Further coding entailed evaluation of the retrieved videos to decipher the intention of the uploaders to the public audience. All relevant videos retrieved from all channels were watched and scrutinized to categorize the videos’ intention to the audience. Videos were categorized based on the messages they conveyed, along with the presenters’ acts, tone, role playing, and clothing (where videos were available). For sexuality-related videos, we analyzed the video based on how the presenter (s) appeared in terms of clothing, gestures made, and acting for visual videos to determine whether the content was sexually explicit. For audio videos, we analyzed the sound background to determine if a video qualified as sexually explicit content. Overall, six categories to code the intention of the visual and/or audio videos were created (see Table 1).

**Table 1:** Categories generated from the videos.

Category	Meaning
Public education	The channel comprised videos in which presenters educated the public about sexuality and SRH^a^. Presenters did not use their personal experiences as examples, but instead backed up their presentations with scientific evidence about the topics they were discussing
Self-promotion or marketing	The channel comprised graphic videos in which the narrators publicized themselves on their sexual techniques to please clients and invited potential clients to visit them for sexual pleasure (involving soft pornographic acting and graphic language)
Predominantly self-promotion with some content about public education	The channel comprised predominantly graphic videos and a few videos that provided information about sexuality and SRH
Sexually explicit content under the disguise of entertainment	The channel comprised movies involving graphic acting around certain issues such as extramarital affairs, male erectile dysfunction, premature ejaculation, and sexual craving among youth
Sexually explicit content under the disguise of sexual education	The channel had graphic videos in which narrators dressed in a sexually revealing manner and used graphic language. Narrators in the video claimed to be educating couples on sexuality but they were using obscene Kinyarwanda words
Predominantly public education with some content about self-promotion	The channel had videos in which narrators provided education about sexuality to couples using culturally acceptable Kinyarwanda words

^a^SRH: sexual and reproductive health.

To explore sexuality and SRH topics covered by the channels from the retrieved videos, a checklist rubric for the visual and audio video analysis was designed to report the themes that were covered by the channels. This checklist was developed after watching the first 20 videos. The checklist was refined if a new message and/or concept had emerged from the retrieved videos. The final checklist with 37 items was achieved after analyzing both audio and visual videos from 100 channels, and then the videos retrieved from eligible channels were critically and rigorously analyzed by three members of the research team (TCU, MGSM, RN) to assess the presence of concepts identified in the checklist and validate the checklist. Data were collected and recorded in the created SPSS data set.

### Data Analysis

Data were analyzed using SPSS Statistics Version 25.0 (IBM, Armonk, NY, USA). Descriptive statistics comprising frequency and percentages were computed to characterize the basic characteristics of retrieved channels, portrayal of the videos, and presentation of sexuality and reproductive health themes that emerged from the retrieved videos. Further analysis involved the computation of cross-tabulations to explore if there were any associations between: (1) focus of the channel and the date when the channel was opened and (2) focus of the channel and who was involved in the video.

To achieve this analytical plan, we collapsed the date when channels were opened as follows: channels that were opened between January 1, 2010, and March 14, 2020, were categorized under “before the COVID-19 pandemic” and channels opened after this period (March 15, 2020, to January 25, 2022) were categorized under “channels that were opened during the COVID-19 pandemic.” This manipulation was done to explore the influence of COVID-19 on the communication of sexuality and reproductive health on Kinyarwanda YouTube channels.

### Ethical Considerations

The institutional review board of the University of Rwanda, College of Medicine and Health Sciences deemed ethics approval unnecessary as the content that was analyzed was in the public domain. Confidentiality and privacy were assured through not revealing the names of the channels and the owners in the reporting of the results. Furthermore, we did not add any video to illustrate the themes presented by the channels because some of the videos were sexually sensitive and in other cases videos involved disclosure cases such as sexual and gender-based violence, child abuse, and acts of indecent assault.

## Results

### Search Results

The search of YouTube yielded a total of 1369 channels comprising 112,348 videos at the time of data collection. Among these videos, 21,506 tackled sexuality and SRH topics. The majority of channels (1333/1369, 98.2%) were owned by individuals, followed by private companies (17/1369, 1.3%), professional public organizations and SRH nongovernmental organizations (3/1369, 0.2% for both), and religious institutions (2/1369, 0.1%).

### Characteristics of the Retrieved Channels

Most of the retrieved channels (518/1369, 37.8%) focused predominantly on adult content, followed by channels that covered other topics with sexually explicit videos (292/1369, 21.4%), movie channels (256/1369, 18.7%), entertainment channels (176/1369, 12.9%), health-related channels (69/1369, 5.1%), channels focusing solely on SRH education (40/1369, 2.9%), and religious channels (18/1369, 1.3%). Regarding the time period when the retrieved channels had been operational, 827 (60.4%) had been in existence within at least 2 years because they were opened during the COVID-19 pandemic; 456 (33.3%) were opened between January 2016 and March 14, 2020; and 86 (6.3%) opened between January 2010 and December 2015.

**Figure 1:**
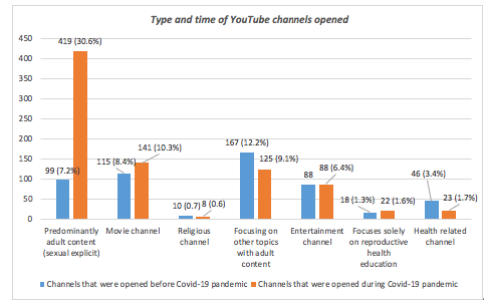
Information about the YouTube channels retrieved.

We examined if there was any association between the type of channel and the date when the channel was opened. As shown in Figure 1, during the COVID-19 pandemic, there was a 4-fold increase (from 7.2% to 30.6%) of the channels that solely focused on sexually explicit content (eg, presenters dressed in sexually revealing clothes, talking or acting in a way suggesting soft pornography, nudity, speaking of personal sexual experience in graphic detail, speaking about sexual performance in graphic detail). The *χ*^2^ test showed that this association between the increase of sexually explicit channels and COVID-19 was statistically significant (*P*<.001). Furthermore, we found that 38 channels opened before 2020 actively uploaded sexually explicit content during the COVID-19 pandemic period (from March 15, 2020, to January 25, 2022).

Regarding the subscription trend, 722 (52.7%) of the 1369 channels had recorded no subscribers and 647 (47.3%) recorded between 1 and 5,000,000 subscribers. As for the number of viewers, the majority of channels (n=1260, 92.8%) had between 1 and 1 million viewers and only 98 channels (7.2%) had over 1 million viewers.

Viewers’ protection was examined in the way the channels forewarn viewers of any videos that may appear inappropriate for certain members of the audience, the type of warning the channels set, and if the content uploaded is expert- and/or evidence-based. Most of the channels (1083/1369, 79.2%) did not warn viewers if they uploaded content that was sensitive, 111 (8.1%) channels inconsistently warned viewers, and only 6 (0.4%) channels warned viewers of any inappropriate content uploaded on the channels. In 169 of the 1369 channels (12.3%), there was no need to warn viewers. As for the acceptability of the language used in the videos, most of the channels (788/1369, 57.6%) used dirty and slang sexually explicit language in the context of the Kinyarwanda language, 259 channels (18.9%) had used formal Kinyarwanda language in the video (if the content was graphic, they found a euphemistic way to present it in culturally acceptable Kinyarwanda language), and 322 channels (23.5%) had combined both culturally acceptable and sexually explicit language. Only 51 channels (3.7%) had videos in which the messages were provided by an expert and/or a trained professional in the fields under study.

### Characteristics of the Retrieved Videos

The characteristics of the videos are summarized in Table 2 and the narrator sex distribution for each area of focus of the videos is presented in Table 3. The majority of the 1369 channels (n=876, 64%) involved video interview and acting. Most of the channels (515/1369, 37.6%) posted a video related to sexuality and/or SRH at least every month. Most of the channels (784/1369, 57.3%) solely involved females. Cross-tabulation analysis indicated that 29.7% (n=406) of the videos from channels that only focused on adult content involved females.

**Table 2:** Information about the videos among the retrieved YouTube channels (N=1369).

Characteristic		Channels, n (%)
**Video type**		
	Video interviews	127 (9.3)
	Audio talk	208 (15.2)
	Both video and audio content (a channel having some video interviews and some audio talks recorded)	151 (11.0)
	Video talk and acting	876 (64.0)
	Audio talk and acting	7 (0.5)
**Posting timeline interval**		
	The channel was opened recently	45 (3.3)
	Between 1 day and 3 days	12 (0.9)
	Every week	312 (22.8)
	Every month	515 (37.6)
	Every 2 months	239 (17.5)
	Between 3 and 12 months	179 (13.1)
	Over 1 year	67 (4.9)
**Sex of the individuals involved in the video**		
	Male	118 (8.6)
	Female	784 (57.3)
	The video involves males and females talking	465 (34.0)
	Child	2 (0.1)

**Table 3:** Distribution of narrator sex according to the focus of the YouTube channel (N=1369).

Focus of the channel	Male, n (%)	Female, n (%)	Male and female, n (%)	Child, n (%)	Total, n (%)
Predominantly adult content (sexually explicit)	16 (1.2)	406 (29.7)	95 (6.9)	1 (0.1)	518 (37.8)
Movie	6 (0.4)	70 (5.1)	180 (13.1)	0 (0)	256 (18.7)
Religion	4 (0.3)	7 (0.5)	7 (0.5)	0 (0)	18 (1.3)
Other topics with adult content	38 (2.8)	159 (11.6)	95 (6.9)	0 (0)	292 (21.3)
Entertainment	10 (0.7)	102 (7.5)	63 (4.6)	1 (0.1)	176 (12.9)
Solely on sexual and reproductive health education	14 (1.0)	18 (1.3)	8 (0.6)	0 (0)	40 (2.9)
Health-related topics	30 (2.2)	22 (1.6)	17 (1.2)	0 (0)	69 (5.0)
Total	118 (8.6)	784 (57.3)	465 (34.0)	2 (0.1)	1369 (100.0)

Concerning the intention of the video to the audience, 700 (51.1%) of the 1369 channels contained self-promotion videos with sexually suggestive attire, nudity, or acting and transmitting the message in graphic way; 233 (17.1%) covered sexually explicit content under the disguise of entertaining the viewers (eg, movies in which acting around certain issues such as extramarital affairs and issues around male erectile dysfunction or premature ejaculation was graphic); and 253 (18.5%) contained sexually explicit content under the disguise of educating the public. Only 183 channels (13.4%) intended to educate the public about different topics of sexuality and SRH.

### Themes Conveyed by the Retrieved Videos

#### Overall Themes

The majority of retrieved channels (1150/1369, 84.0%) tackled the topic of sexuality, with sexually explicit content predominantly found in most of these videos (n=1082, 79%), and only 287 (16%) videos covered SRH matters.

Themes that emerged from retrieved videos were grouped into two categories: (1) those that focus on sexuality and (2) those that focus on reproductive health components.

#### Focus on Sexuality

Sexuality themes encompassed videos that focused on sexual intercourse performance; promotion of sex work; and tips to enhance sexual practice to achieve erotic pleasure, including techniques and roles of traditional practices in leveraging sexual performance (Table 4) and issues affecting sexual intercourse performance (Table 5).

Three quarters of channels (969/1369, 70.8%) had videos that promoted sex work and prostitution; 859 channels (62.7%) had uploaded videos that focused on how to make a female squirt during sexual intercourse (*kunyaza* practice); 818 (59.8%) channels had videos that taught viewers about sex positions and techniques to maximize pleasure during intercourse; 582 (42.5%) promoted unprotected sexual intercourse; 356 channels (26.0%) taught about oral sex (fellatio) and anal sex; and 228 (16.7%) channels, mostly movie channels, had content that incited young people to engage in sex and promiscuity. In addition, 356 (26%) channels had videos that focused on the types of female vaginas based on their shapes and positions and how men can handle any of these vaginas during sex to achieve mutual maximum pleasure. Equally, 220 (16.1%) channels had videos about the types of penises based on their length and shape and how they are used to enhance female pleasure (Table 4).

As shown in Table 5, most channels (66.5%) had videos that discussed techniques to prepare the female partner for sex. Over half of the videos (55.3%) focused on secrets to achieve female sexual pleasure and orgasm. Almost half of the channels (49.6%) covered videos about tricks and techniques women should use to please men in bed. Only a minority of channels (19.1%) had videos that promoted the benefits of the labia stretching (*gukuna*), a cultural sexual practice in Rwanda.

**Table 4:** Nature of YouTube channels focusing on sexual intercourse performance and promotion of sex work (N=1369).

Focus		Channels, n (%)
**Positions and techniques to make love with role-play demonstrations and how the demonstrated position lead to the maximization of both partners’ sexual pleasure**		
	Yes	818 (59.8)
	No	551 (40.2)
**The role of female labia elongation (*gukuna*) on vaginal moisture during sexual intercourse**		
	Yes	407 (29.7)
	No	962 (70.3)
**Sexual intercourse involving same-sex couples**		
	Yes	84 (6.1)
	No	1285 (93.9)
**Female squirting during intercourse from the Rwandan cultural context (*kunyaza*)**		
	Yes	859 (62.7)
	No	510 (37.3)
**Sex involving the use of the mouth and anus for both male and female partners**		
	Yes	356 (26.0)
	No	1013 (74.0)
**Sex during pregnancy: positions and precautions to take by the male partners**		
	Yes	148 (10.8)
	No	1221 (89.2)
**Promoting unprotected sexual intercourse**		
	Yes	582 (42.5)
	No	787 (57.5)
**Promotion of sex work and prostitution (sexually explicit acting, talking, and dressing; how sex work is done; who is involved; gains; and dangers)**		
	Yes	969 (70.8)
	No	400 (29.2)
**Inciting young people to engage in sex and other promiscuous relationships**		
	Yes	228 (16.7)
	No	1141 (83.3)
**Types of vaginas based on their shapes and how male partners can handle them during intercourse to maximize the female partner’s sexual pleasure**		
	Yes	356 (26.0)
	No	1013 (74.0)
**Types of male sex organs based on their shape and length and how they can be used to maximize pleasure for the female partner during intercourse**		
	Yes	220 (16.1)
	No	1149 (83.9)

**Table 5:** Nature of YouTube channels related to enhancing sexual pleasure (N=1369).

Focus			Channels, n (%)
**Secrets to achieving female sexual pleasure and orgasm**			
	Yes	757 (55.3)	
	No	612 (44.7)	
**How to please men in bed**			
	Yes	679 (49.6)	
	No	690 (50.4)	
**How to perform well and last long in bed for men (exercises to do, techniques to apply, and medicine to take)**			
	Yes	221 (16.1)	
	No	1148 (83.9)	
**Tips to prepare the female partner for sexual intercourse**			
	Yes	911 (66.5)	
	No	458 (33.5)	
**The role of male circumcision as a hygienic practice, prevention of sexually transmitted infections, and in increasing female sexual pleasure**			
	Yes	227 (16.6)	
	No	1142 (83.4)	
**Promotion of labia elongation (*gukuna* or *guca imyeyo*): sexual benefits, female advertising themselves, individuals or companies marketing products for labia stretching**			
	Yes	261 (19.1)	
	No	1108 (80.9)	

As shown in Table 6, approximately one-third of the channels (30.5%) had videos that focused on masturbation practice, including its benefits, effects on sexual performance, and its treatment, and 280 channels (20.5%) had videos that discussed male premature ejaculation, including its causes, how it affects sexual performance, and how to prevent its occurrence. Moreover, 255 channels (18.6%) had videos that discussed how extramarital affairs occur and how they paralyze couples’ sexual relationships, and 141 (10.3%) channels had videos that promoted “sugar mummies” and “sugar daddies.”

**Table 6:** Nature of YouTube channels addressing issues affecting sexual performance in the Rwandan context (N=1369).

Focus		Channels, n (%)
**Masturbation (both self or by partner, effects, benefits, causes, and herbs and/or other treatments to stop masturbation)**		
	Yes	418 (30.5)
	No	951 (69.5)
**Male premature ejaculation (its causes, how it may affect female and male partners’ sex interactions, tips including food and exercise to prevent it, and treatment)**		
	Yes	280 (20.5)
	No	1089 (79.5)
**Tips to enhance and maximize sexual pleasure among heterosexual married or cohabitating couples**		
	Yes	206 (15.0)
	No	1163 (85.0)
**Extramarital affairs (causes, advice for and against the practice, negative consequences associated with the practice, and solutions)**		
	Yes	255 (18.6)
	No	1114 (81.4)
**Promotion of prostitution with sugar mummies and/or sugar daddies**		
	Yes	141 (10.3)
	No	1228 (89.7)

#### Focus on Reproductive Health

Themes about reproductive health encompassed SHR education, as summarized in Table 7 and Table 8.

Sexual and reproductive health education was covered in 57 (4.2%) channels that had videos about sexually transmitted diseases, including HIV/AIDS. Among the 1369 channels, 95 (6.9%) uploaded videos that provided advice to young people about enhancing their reproductive health, 98 (7.2%) channels had videos that covered topics about condom (mis) use to prevent sexually transmitted infections and/or unwanted pregnancies, and only 41 (3.0%) channels had videos about contraceptive use to prevent unwanted and/unplanned pregnancies (Table 7).

As shown in Table 8, 96 (7.0%) channels had videos that taught women about their reproductive cycle and how they should manage this period, and 59 (4.3%) channels had videos on what the woman needs to ensure for safe pregnancy, childbirth, and protecting the unborn baby from harm. As for abortion, 45 (3.3%) channels had videos that reported about safe and unsafe abortions, including how it is performed, consequences, and postabortion care offered to those affected.

**Table 7:** Nature of YouTube channels focusing on sexual and reproductive health education (N=1369).

Focus			Channels, n (%)
**Sexually transmitted diseases, including HIV/AIDS (symptoms, diagnosis, treatment, and prevention)**			
	Yes	57 (4.2)	
	No	1312 (95.8)	
**Advice about reproductive health for young people (abstinence, protection, learning to say no to sexual intercourse, and responsible use of social media among youth)**			
	Yes	95 (7.0)	
	No	1274 (93.0)	
**The meaning of the concept of sex from the perspectives and context of young and adult people who are in a love relationship**			
	Yes	175 (12.8)	
	No	1194 (87.2)	
**Food to eat to enhance male stamina during sex and to boost sperm count**			
	Yes	48 (3.5)	
	No	1321 (96.5)	
**Advice to ensure good reproductive health for both men and women (food, exercise, screening, good mental health)**			
	Yes	50 (3.7)	
	No	1319 (96.3)	
**Condom (mis) use to prevent sexually transmitted infections or unwanted/unplanned pregnancies**			
	Yes	98 (7.2)	
	No	1271 (92.8)	
**Contraceptive use to prevent unwanted and/or unplanned pregnancies (medicines used, benefits, how they are used, side effects)**			
	Yes	41 (3.0)	
	No	1328 (97.0)	

**Table 8:** Nature of YouTube channels focused on women’s reproductive health care (N=1369).

Focus			Channels, n (%)
**Female reproductive cycle (menstruation, ovulation, and safe days) and how to behave during the menstrual cycle**			
	Yes	96 (7.0)	
	No	1273 (93.0)	
**Hygiene of female sexual organs (for purposes of male sexual pleasure, during periods, and fighting off infections and bad smell)**			
	Yes	93 (6.8)	
	No	1276 (93.2)	
**What to do to ensure safe pregnancy and childbirth and tips to protect the unborn baby from harm**			
	Yes	59 (4.3)	
	No	1310 (95.7)	
**Abortion (both safe and unsafe abortions, consequences, and support provided to those affected)**			
	Yes	45 (3.3)	
	No	1324 (96.7)	

## Discussion

### Principal Findings

Our study provides evidence about YouTube utilization to communicate sexuality and reproductive health issues in Rwanda, the characteristics of the Kinyarwanda YouTube channels, and aspects of SRH covered by these channels.

The results suggest varying levels of interest and curiosity among the audience regarding different aspects of sexual pleasure, techniques, practices, and gender-specific topics. These findings can inform the development of educational materials, campaigns, and interventions related to sexual health and pleasure to address the specific needs and interests of the target audience.

The results show that during the COVID-19 pandemic, there has been a proliferation of Kinyarwanda YouTube channels. This rise in YouTube channels may be tracked to the lockdowns that were imposed to contain the spread of the pandemic that led to some people’s jobs being suspended or even terminated [19-21]. Since YouTube can be used to generate profit from uploaded individual videos [22], it is possible that some individuals have created channels covering sexuality-related issues, entertainment, and movies to attract views and subscriptions from viewers who needed entertaining distractions to enable them to cope with the negative effects and stressors of the COVID-19 pandemic [23] and the consequences resulting from reduced social contacts [24]. More particularly, we found that during the COVID-19 pandemic, there was a high increase of channels dealing with sexually explicit content in Kinyarwanda, as our findings note that 419 YouTube channels were opened during this period. We could not establish the reasons to interpret this increase; hence, further research is needed to understand, from the channel owners’ perspectives, why adult content became the predominant topic during the COVID-19 pandemic.

This study found that over half of the channels had no subscribers. This can be viewed from the perspective of Rwanda’s cultural context that often disparages the open discussion of sexuality-related matters [25]. It is possible that visitors of the channels fear subscribing to them so as to not diverge from the socially accepted practices of Rwanda, although they viewed the content of the channels. Another reason to explain this phenomenon is that some of the channels were only recently opened, which means that people may not have yet been aware of them.

Our study found that most of the channels did not warn viewers about videos that used sexually explicit and obscene language, which is surprising in consideration of Rwandan culture. We anecdotally assumed that pressure to obtain more views and likes without considering the harms the videos may cause among some audiences such as teenagers and children can explain why the majority of the channels with offensive content do not protect viewers, especially channels including videos talking about sexual intercourse and pleasure. Another possible reason to explain this may be the complexity in regulation of social media use in Rwanda.

We found that most of the videos from channels that only focused on adult content involved females. Based on the videos watched, we suggest that the channel owners sexually objectify females for their own interests [26]. Thus, channels containing videos in which females are discussing sex matters while dressed in sexually revealing attire may attract more views, likes, subscriptions, and shares. However, we cannot confirm this speculation. Hence, further research is needed to understand the motives behind the increased number of videos involving more women than men discussing sexuality-related issues in the Rwandan context.

Over half of the videos focused on secrets to achieve female sexual pleasure and orgasm. This suggests a need for education and information on this topic, indicating a potential knowledge gap or curiosity among the audience. Further research is needed to determine women’s satisfaction of sexual intercourse and factors predicting women’s achievement of orgasm during sexual encounters within the Rwandan community.

Almost half of the channels contained videos that focused on teaching women how to please men in bed. This highlights the interest in understanding and improving sexual experiences for men, indicating the perceived importance of satisfying their partners. Surprisingly, only a minority of channels focused on teaching techniques, exercises, and medicine to enhance sexual performance for men. Compared to the previous two topics, this finding suggests that men are under less pressure when it comes to satisfying sexual relationships among couples, with the majority of the pressure on women to please their male partners. By contrast, our study found that a significant majority of videos focused on tips for preparing the female partner for sexual intercourse. This indicates the importance placed on ensuring the comfort and readiness of the female partner, highlighting a concern for mutual satisfaction and well-being. Therefore, further research is needed to understand, from both men’s and women’s perspectives, the factors affecting satisfying sexuality in Rwanda.

A relatively small proportion of videos from the retrieved channels reported the role of male circumcision in hygiene, sexually transmitted infection prevention, and female sexual pleasure. This suggests a potential need for more education and information about the benefits and implications of male circumcision in relation to sexual health.

Compared to sexuality-related topics, there were very few videos covering reproductive health topics such as preconception health, pregnancy, childbirth, family planning, sexually transmitted infections, menstruation, abortion, and adolescent reproductive health. This finding corroborates the results of a previous study by Kinsler et al [27], demonstrating that narratives on television about sexuality and SRH education in the United States are lacking educational information about low-risk behaviors.

The available videos in which these reproductive health topics had been discussed adequately belonged to religious and nongovernmental channels. There is still low involvement of individual YouTube owners in focusing on reproductive health matters.

### Limitations

This study has some limitations. First, this study aimed at describing the available information about SRH in the Kinyarwanda language. We did not assess the quality and authenticity of the information transmitted in the retrieved videos. Although our search strategy was extensive, we may have missed videos that were not properly tagged with matching search terms. In addition, some videos that were retrieved in our study may have been deleted since the time of our analysis.

Although we examined the trend of SRH communication during the COVID-19 pandemic, we did not perform a longitudinal analysis of the videos over an extended period. Therefore, further research is needed to understand the changes and developments in sexuality and SRH communication on YouTube over time to provide valuable insights.

The coding process for our study involved subjective evaluation of videos to categorize their content and intention. Different researchers may interpret the videos differently, leading to potential bias or inconsistencies in the coding scheme. In addition, our study could not explain intercoder reliability to confirm that the coding results were unbiased. Finally, as we retrieved publicly available content, we were only able to analyze the content available from Kinyarwanda-language YouTube videos obtained. We did not ask viewers their motivations for watching videos or the impact of these videos on their sexuality and reproductive health behaviors. We also did not ask the owners of the retrieved YouTube channels about their motivations for uploading videos and how they assess the effects of their videos on the public. Lastly, our study focused on analyzing the videos themselves and did not gather information about the viewers of these videos. Therefore, further research is needed to understand the demographics and characteristics of the viewers to provide valuable insights into the impact and effectiveness of sexuality and SRH communication on YouTube.

### Conclusion

This is the first study to analyze the use of YouTube in communicating about sexuality and reproductive health in the Kinyarwanda language. This study provides a snapshot on how Kinyarwanda YouTube channels discuss sexuality and SRH issues. A high number of Kinyarwanda YouTube channels are covering sexually explicit content and most of these channels appeared during the COVID-19 pandemic. Moreover, channels with sexuality-related videos rarely warn viewers about the videos they are about to watch in case the content may be sensitive. Thus, this study suggests some policy implications. The Rwanda Ministry of Information Communication Technology and Innovation should work collaboratively with the Ministries of Culture and Gender and Family Promotion to devise ways of regulating and monitoring the content uploaded on Kinyarwanda YouTube channels. The Ministry of Health and other stakeholders engaged in promoting SRH need to leverage the use of social media, especially YouTube, in their SRH interventions so that they reach more people, especially young people. This study relied on the videos that appeared online during the study period. Finally, since our study did not gather information about the perceptions and perspectives of channel owners and individuals involved in creating the videos, we suggest that further research be done to understand their motivations, intentions, and perceived impact on the Rwandan community’s sexuality and SRH in order to provide a more comprehensive understanding of the content and its implications. Further research should gather information about who accesses the videos, and how channel owners and individuals involved in the videos perceive the impact of their videos on the Rwandan community’s sexuality and reproductive health well-being.

## Abbreviations

SRH: sexual and reproductive health

## Data Availability

The data sets generated during and/or analyzed during this study are available from the corresponding author on reasonable request.
